# Differential expression of stem cell markers in proliferating cells in glioma

**DOI:** 10.1007/s00432-021-03704-5

**Published:** 2021-06-25

**Authors:** Marten Rehfeld, Jakob Matschke, Christian Hagel, Kerstin Willenborg, Markus Glatzel, Christian Bernreuther

**Affiliations:** 1 Institute of Neuropathology, University Medical Center Hamburg-Eppendorf, Martinistrasse 52, 20246 Hamburg, Germany; 2 Department of Otolaryngology, Head and Neck Surgery, Hannover Medical School, Hannover, Germany; 3 Institute of Pathology, University Medical Center Hamburg-Eppendorf, Hamburg, Germany

**Keywords:** Glioma, Glioblastoma, Stem cells, Ki67, Proliferating cells, Expression profile

## Abstract

**Purpose:**

The identification of prognostically and therapeutically relevant molecular markers is fundamental to the further development of personalised therapies in brain tumours. Current therapeutic options for the treatment of gliomas rely mainly on surgical resection and the inhibition of tumour cell proliferation by irradiation and chemotherapy. Glioma stem cells are a subpopulation of proliferating tumour cells that have self-renewal capacity and can give rise to heterogeneous cells that comprise the tumour and are thought to play a role in the resistance of gliomas to therapy. The aim of this study was to evaluate the expression of markers of glioma stem cells and differentiated glial cells in proliferating glioma cells in comparison to the overall expression of the respective markers in the tumour tissue.

**Methods:**

Tissue microarrays were assembled from specimen of pilocytic astrocytoma, diffuse astrocytoma, anaplastic astrocytoma, glioblastoma, oligodendroglioma, anaplastic oligodendroglioma, ependymoma, and anaplastic ependymoma. These were immunohistochemically double stained with antibodies against the proliferation-associated antigen Ki67 and marker proteins for glioma stem cells (CD133, Nestin, Musashi, CD15, CD44), and differentiated glioma cells (GFAP, MAP2c).

**Results:**

The expression of both glial and glioma stem cell markers differs between proliferating and non-proliferating glioma cells. Furthermore, the proliferating cells in the different glial tumour entities show a different expression profile.

**Conclusion:**

Further analysis of marker expression in proliferating glioma cells and correlation with clinical outcome and susceptibility to irradiation and chemotherapy might help establish new biomarkers and therapies for glioma.

## Introduction

Along with metastases gliomas are the most prevalent tumours in the adult human brain. While some primary brain tumours like pilocytic astrocytoma, ependymoma, and ganglioglioma often follow a benign course, most glial tumours share a poor prognosis. This holds especially true for glioblastoma, the most prevalent and malignant primary brain tumour with a median survival time of 15 months (Stupp et al. 2014). The estimation of prognosis and the choice of therapy depend on the histopathologic classification and grading according to the World Health Organization (WHO) classification of tumours of the central nervous system but success of therapy, progression-free and overall survival vary even among tumours of the same entity and grade. Therefore, the identification of biomarkers correlating with prognosis and the susceptibility to therapy is pivotal to personalized therapy. On the genetic level, 1p/19q codeletion, methylation of the promoter of O^6^-methylguanin-methyltransferase (MGMT), and point mutations in the gene encoding isocitrate dehydrogenases (IDH) 1 und 2 have been established as molecular markers in the diagnosis of brain tumours that guide therapeutic decisions (Louis, 2014; Weller et al. 2012).

Current therapeutic options comprise surgical resection, irradiation, chemotherapy, and inhibition of angiogenesis. Thus, non-surgical therapy mainly targets proliferating cells. Many studies demonstrated a correlation between proliferation and prognostic factors in astrocytic tumours but proliferation as a solitary marker did not allow reliable predictions in single cases (Johannessen and Torp 2006). The identification of glioma stem cells, a subpopulation of tumour cells that have self-renewal capacity and can give rise to heterogeneous tumour cells are thought to play a role in the resistance of glioblastoma to therapy (Singh, 2004). Glioma stem cells share properties with neural stem cells and may serve as new targets for therapy by selective inhibition of proliferation or the induction of differentiation of glioma stem cells (Chen et al. 2012). Glioma stem cells are operationally defined by their ability to form spheres in cell culture and to lead to tumour formation after transplantation in animal models. Furthermore, molecular markers have been identified that are expressed by glioma stem cells. For some of these marker proteins like CD133 and Musashi-1 a correlation between their expression and other prognostic factors could be shown (Dahlrot et al. 2013; Pallini, 2008), but systematic studies analysing co-expression of different marker proteins on a single cell level have to the best of our knowledge not been performed.

Because most non–surgical therapeutic options for glioma target proliferating cells and considering the potential role of glioma stem cells in the resistance of glioblastoma to therapy, the aim of this study was to immunohistochemically characterise the marker expression of proliferating cells in glioma focussing on markers expressed by differentiated glioma cells and glioma stem cells in comparison with marker expression in non-proliferating cells.

## Materials and methods

### Tissue

**Fig. 1:**
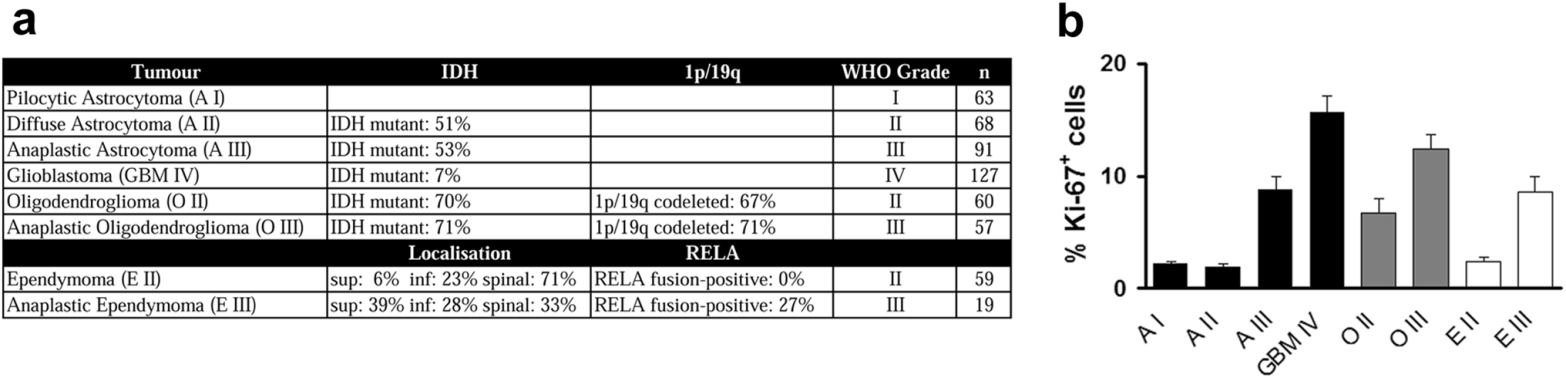
Characterisation of the tissue microarrays (TMAs). **a** Specification of the tissue used for the generation of the tissue microarrays (TMA). Tumour entity with respective WHO grade and numbers of the specimen (*n*) are shown. For diffuse and anaplastic astrocytoma and glioblastoma, the percentage of isocitrate dehydrogenase (IDH) mutant tumours as detected by immunohistochemical analysis with antibodies against mutant IDH1 carrying the R132H mutation is shown. For oligodendroglioma and anaplastic oligodendroglioma the percentage of tumours carrying the 1p/19q codeletion as detected by fluorescence in situ hybridisation is shown, additionally. For ependymomas and anaplastic ependymomas the percentages of tumours in supratentorial (sup), posterior fossa/infratentorial (inf), and spinal location is shown. Furthermore, the percentage of RELA fusion-positive ependymomas is shown. **b** Analysis of proliferation in pilocytic astrocytoma WHO grade I (A I), diffuse astrocytoma WHO grade II (A II), anaplastic astrocytoma WHO grade III (A III), glioblastoma WHO grade IV (GBM IV), oligodendroglioma WHO grade II (O II) and III (O III), ependymoma WHO grade II (E II), and anaplastic ependymoma WHO grade III (E III). Mean percentages of Ki-67-positive cells of all cells ± s.e.m. are shown

Formalin-fixed paraffin-emdebbed tissue of 63 pilocytic astrocytomas WHO grad I, 68 diffuse astrocytomas WHO grad II (IDH mutant: 51%, NOS: 49%), 91 anaplastic astrocytomas WHO grade III (IDH mutant: 53%, NOS: 47%), 127 glioblastomas WHO grad IV (IDH mutant: 7%, IDH wild-type: 49%), 60 oligodendrogliomas WHO grad II (IDH mutant: 70%, 1p/19q codeleted: 67%, NOS: 33%), 57 anaplastic oligodendrogliomas WHO grad III (IDH mutant: 71%, 1p/19q codeleted: 71%, NOS: 29%), 59 classic ependymomas WHO grad II (supratentorial: 6%, infratentorial: 23%, spinal: 71%, RELA fusion-positive: 0%, no papillary, myxopapillary, clear cell or tanycytic ependymomas), and 19 anaplastic ependymomas WHO grade III (supratentorial: 39%, infratentorial: 28%, spinal: 33%, RELA fusion-positive: 27%, no papillary, myxopapillary, clear cell or tanycytic ependymomas) diagnosed between 1981 and 2013 were retrieved from the archive of the Institute of Neuropathology of the University Medical Center Hamburg-Eppendorf in accordance with local laws and legislation (Fig. 1a). Diagnoses were confirmed and tumours were classified following the 4^th^ edition of the WHO classification of tumours of the central nervous system (Louis et al. 2016). To this end, the IDH mutation status was determined by immunohistochemistry with an antibody against mutant IDH1 carrying the R132H mutation and the 1p/19q status was determined by fluorescence in situ hybridisation (FISH). Furthermore, an immunohistochemical analysis with an antibody against NF-κB p65 (p65) was performed to detect RELA fusion-positive ependymomas. All analyses were performed on the tissue microarrays (TMAs). TMAs were assembled as described (Kallioniemi et al. 2001; Kononen, 1998). Briefly, histological slides stained with hematoxylin and eosin (H&E) were reviewed and representative tumour areas were marked on the slide and the corresponding paraffin block. For the assembly of the TMAs, tissue cores of 0.6 mm diameter were acquired from the paraffin blocks of the original tumour samples by a thin-walled needle and were assembled in a new block. The use of archived remnants of diagnostic tissues for manufacturing of TMAs and their analysis for research purposes as well as patient data analysis has been approved by local laws (HmbKHG, §12) and by the local ethics committee (Ethics commission Hamburg, WF-049/09). All work has been carried out in compliance with the Helsinki Declaration.

### Immunohistochemistry

4-μm-thick sections were deparaffinised and submitted to immunostaining using the Ventana Benchmark XT machine (Ventana Medical Systems, Tucson, AZ, USA). Primary antibodies used were Mouse monoclonal antibodies against CD15 (1:100; 559,045, BD Biosciences, Heidelberg, Germany), CD44 (1:50; M7085, Dako, Glostrup, Denmark), CD68 (1:250; M0876, Dako), CD133 (clone 17A6.1) (1:100; MAB4399, Millipore, Temecula, CA, USA), epithelial membrane antigen (EMA) (1:100; M0613, Dako), glial-fibrillary acidic protein (GFAP) (1:100; M0761, Dako), Ki-67 (clone SP6) (1:50; RM-9106-S; Neomarkers, Fremont, CA, USA), IDH1 R132H (1:20; DIA-H09, Dianova, Hamburg, Germany) leucocyte common antigen (LCA) (1:400; M0701, Dako), microtubulus associated protein 2 (MAP2c) (1:400; M4403, Sigma, Deisenhofen, Germany), and Nestin (clone 10c2) (1:50; sc-23927; Santa Cruz), rabbit polyclonal antibodies against Musashi-1 (1:200; AB5977, Millipore), and rabbit monoclonal antibodies against p65 (1:2000; #8242, Cell Signaling, Danvers, MA, USA) diluted in 5% goat serum (Dianova), 45% Tris buffered saline pH 7.6 (TBS), 0,1% Triton X-100 in antibody diluent solution (Zytomed, Berlin, Germany). For immunohistochemical staining with a single antibody, Ventana standard staining procedure including pretreatment with cell conditioner 1 for 60 min for antibodies against CD15, CD44, CD68, EMA, GFAP, Ki-67, LCA, MAP2c, Musashi-1, and Nestin was applied whereas no pretreatment was performed for immunohistochemical staining with antibodies against CD133. Primary antibodies were incubated at 37 °C for 32 min followed by Ventana standard signal detection with ultraView Universal Alkaline Phosphatase Red Detection Kit (Ventana Medical Systems) and counterstaining with hematoxylin. For immunohistochemical double staining with antibodies against Ki-67, Ventana standard staining procedure was performed for the respective antibody as described above with the exception that counterstaining with hematoxylin was omitted. Then, Ventana standard staining procedure was applied using antibodies against Ki-67 as described above with the exception that Ki-67 antibodies were visualized with UltraView Universal DAB Detection Kit (Ventana Medical Systems) followed by counterstaining with hematoxylin.

### Fluorescence in situ hybridisation

A commercial paraffin pretreatment reagent kit (Abbott, Chicago, IL, USA) was used for proteolytic pretreatment of freshly cut TMA sections. 4 µm TMA sections were deparaffinised, air-dried, and dehydrated in 70%, 85%, and 100% ethanol, followed by denaturation for 10 min at 95 °C in 70% formamide 2 × SSC solution, followed by proteolytic pretreatment at 37 °C for 6 min. The commercially available FISH probe sets consisted of two dual colour probes: a spectrum-orange labeled 1p36 probe with a spectrum-green labelled 1q25 probe as a reference (Z-2075, ZytoVision, Bremerhaven, Germany) and a spectrum-orange labeled 19q13 probe with a spectrum-green labelled 19p13 probe as a reference (Z-2076, ZytoVision). Hybridization was overnight at 37 °C in a humidified chamber. Slides were subsequently washed and counterstained with 0.2 µmol/L 4’-6-diamidino-2-phenylindole in antifade solution. Stained slides were manually interpreted using a fluorescence microscope with 630 × magnification. 1p/19q codeletion was defined as the presence of both, fewer orange 1p36 signals than green 1q25 signals and fewer orange 19q13 signals than green 19p13 signals in ≥ 60% tumor nuclei.

### Quantitative analyses

For quantification of the immunopositivity after staining with a single antibody, one image covering an area of 0.1 mm^2^ was taken in the centre of each specimen of the TMAs with a Zeiss Axiovert S100 microscope (Carl Zeiss Microscopy, Jena, Germany) and the area immoreactive for the respective marker was determined as a percentage of the total area for each image with the AxioVision software (Carl Zeiss imaging Solutions, Göttingen, Germany).

For quantification of the immunopositivity after double staining the total number of cells positive for the respective marker and Ki-67 was determined in each specimen of the TMAs as a percentage of the total number of Ki-67-positive cells in the same specimen.

### Statistical analysis

All statistical analyses were performed using SPSS statistical software (SPSS, Chicago, Ill, USA). For comparison of means, one-way analysis of variance (ANOVA) followed by Tukey HSD post hoc test was applied. Differences were considered statistically significant if p value was less than 0.05.

## Results and discussion

The aim of this study was to characterise the expression profile of proliferating cells in glioma because these cells are the main target of non-surgical therapy. Considering the glioma stem cell hypothesis proposing a hierarchical organisation of tumour cells in glioma (Hale et al. 2013; Singh et al. 2004), we hypothesised that proliferating cells in glioma will show a different expression profile than non-proliferating tumour cells. Furthermore, stem cell markers might be differentially expressed by proliferating cells in different tumour entities. To analyse marker expression in proliferating and non-proliferating tumour cells, specimen of pilocytic astrocytoma WHO grade I, diffuse astrocytoma WHO grade II, anaplastic astrocytoma WHO grade III, glioblastoma WHO grade IV, oligodendroglioma WHO grade II, anaplastic oligodendroglioma WHO grade III, ependymoma WHO grade II and anaplastic ependymoma WHO grade III were assembled to TMAs to allow simultaneous processing of high sample numbers (Fig. 1a). The cases assembled into the TMAs spanned the years 1981 to 2013. Thus, classification according to the current (4th) edition of the WHO classification of tumours of the central nervous system necessitated the determination of IDH mutations and 1p/19q codeletions in astrocytic and oligodendroglial tumours and the RELA fusion in ependymal tumours. As these analyses were performed on the TMAs, sequencing of the IDH1 and IDH2 gene was not possible and IDH mutations could only be detected by immunohistochemical analysis with an antibody against mutant IDH1 carrying the sequencing of the R132H mutation, the percentage of IDH mutant tumours given in Fig. 1a underestimates the real fraction of IDH mutant tumours among the analysed specimens. Furthermore, not all spots on the TMAs were analysable further decreasing the percentage of IDH mutant tumours. The same holds true for the detection of the 1p/19q codeletion and the RELA fusion thus showing the limitations of using TMAs. While TMAs allow the processing of hundreds of specimens simultaneously using identical conditions and avoid selection bias during analysis because whole cores can be analysed the method holds some limitations. A potential limitation in TMAs is intratumoural heterogeneity if the analysed parameter is not evenly distributed within the tumour. In astrocytic gliomas and especially glioblastoma, intratumoural heterogeneity has been described even on the molecular level (Ren, 2007; Sottoriva, 2013). Intratumoural heterogeneity can be addressed by increasing the number of specimens and the number of cores analysed in each specimen thereby also addressing the problem of non-interpretable cores due to poor immunoreactivity or loss of cores during the staining procedure. In our study, we first analysed the percentage of proliferating cells to validate our TMAs for further analyses. Proliferating cells were identified by immunohistochemical analysis with antibodies against Ki67, an antigen that is routinely used to measure proliferation in brain tumours. Mean proliferation indices, determined as the percentages of Ki67-positive cells in the different glioma entities corresponded to published values (Engelhard et al. 2002; Johannessen and Torp 2006; Kuncova et al. 2009; Louis et al. 2016) for all tumour entities represented on the TMAs (Fig. 1b) thus validating the TMAs for further analyses.

**Fig. 2:**
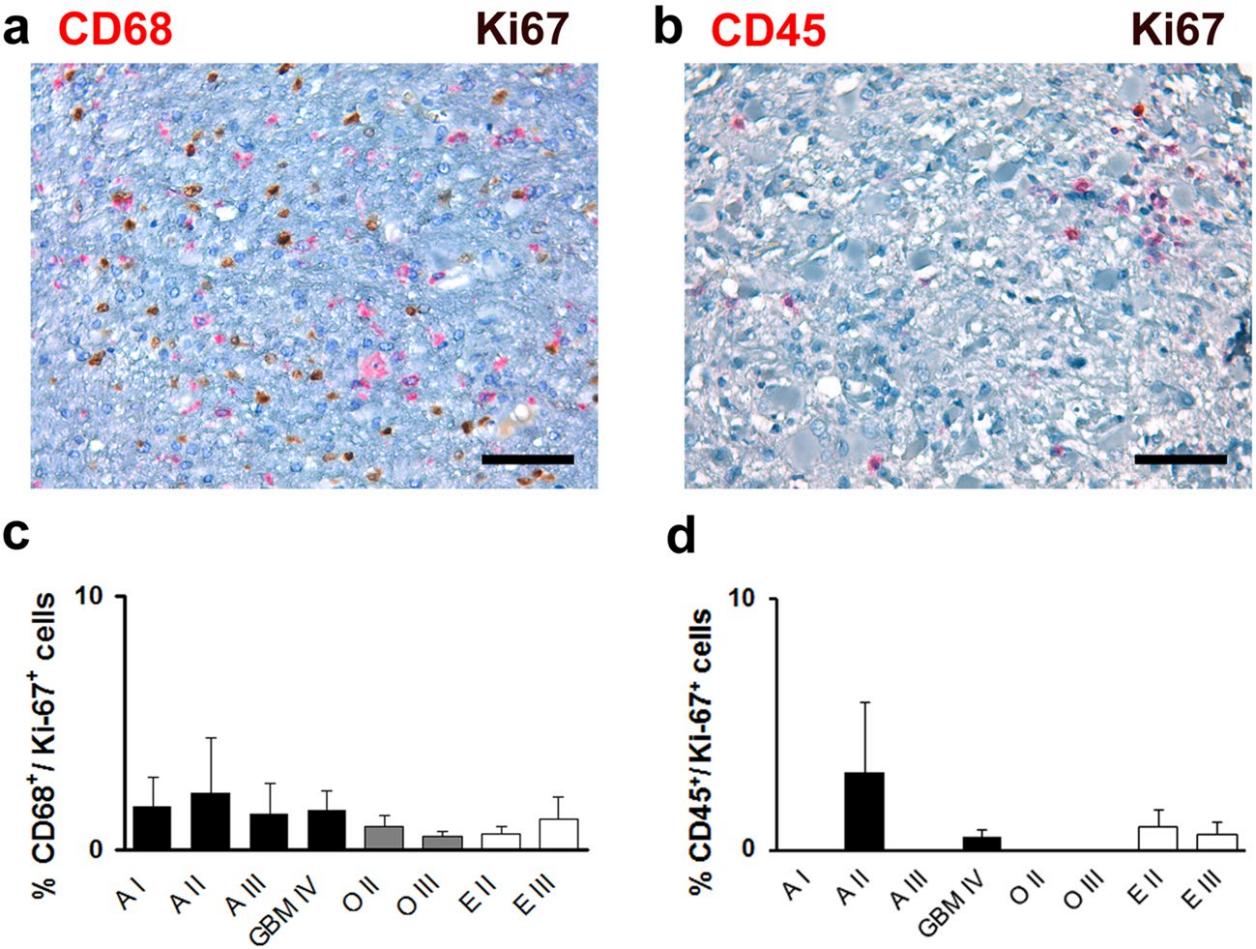
Inflammatory cells account for a low fraction of proliferating cells in glial tumours. Analysis of the expression of inflammatory marker proteins CD68 (**a**, **c**) and CD45 (**b**, **d**) among Ki-67-positive cells in pilocytic astrocytoma WHO grade I (A I), diffuse astrocytoma WHO grade II (A II), anaplastic astrocytoma WHO grade III (A III), glioblastoma WHO grade IV (GBM IV), oligodendroglioma WHO grade II (O II) and WHO grade III (O III), ependymoma WHO grade II (E II), and anaplastic ependymoma WHO grade III (E III). **a** Immunohistochemical double staining with antibodies against Ki-67 (brown) and CD68 (red), scale bar = 100 µm. **b** Immunohistochemical double staining with antibodies against Ki-67 (brown) and CD45 (red), scale bar = 100 µm. **c** Quantification of the percentage of cells immunopositive for both CD68 and Ki-67 among Ki-67 positive cells in glioma. Mean ± s.e.m. are shown. **d** Quantification of the percentage of cells immunopositive for both CD45 and Ki-67 among Ki-67 positive cells in glioma. Mean ± s.e.m. are shown

As lymphocytes and microglia/macrophages have been shown to infiltrate glioma (Hussain et al. 2006; Kuppner et al. 1988), an immunohistochemical analysis with antibodies against Ki67 and CD68 (Supplemental Fig. 2a, c) or CD45 (Fig. 2b, d) was performed showing that the fraction of immune cells among proliferating cells amounted to less than 2% in all tumour entities. Thus, immune cells do not significantly contribute to the pool of proliferating cells in glioma.

To test our hypothesis that proliferating tumour cells in glioma show a different expression profile than non-proliferating cells, immunohistochemical double stainings with antibodies against Ki67 and antigens frequently expressed in glioma or glioma stem cells were performed and percentages of positive cells of all Ki67-positive cells were determined and compared to the total expression of the antigen in the tumour specimen.

**Fig. 3:**
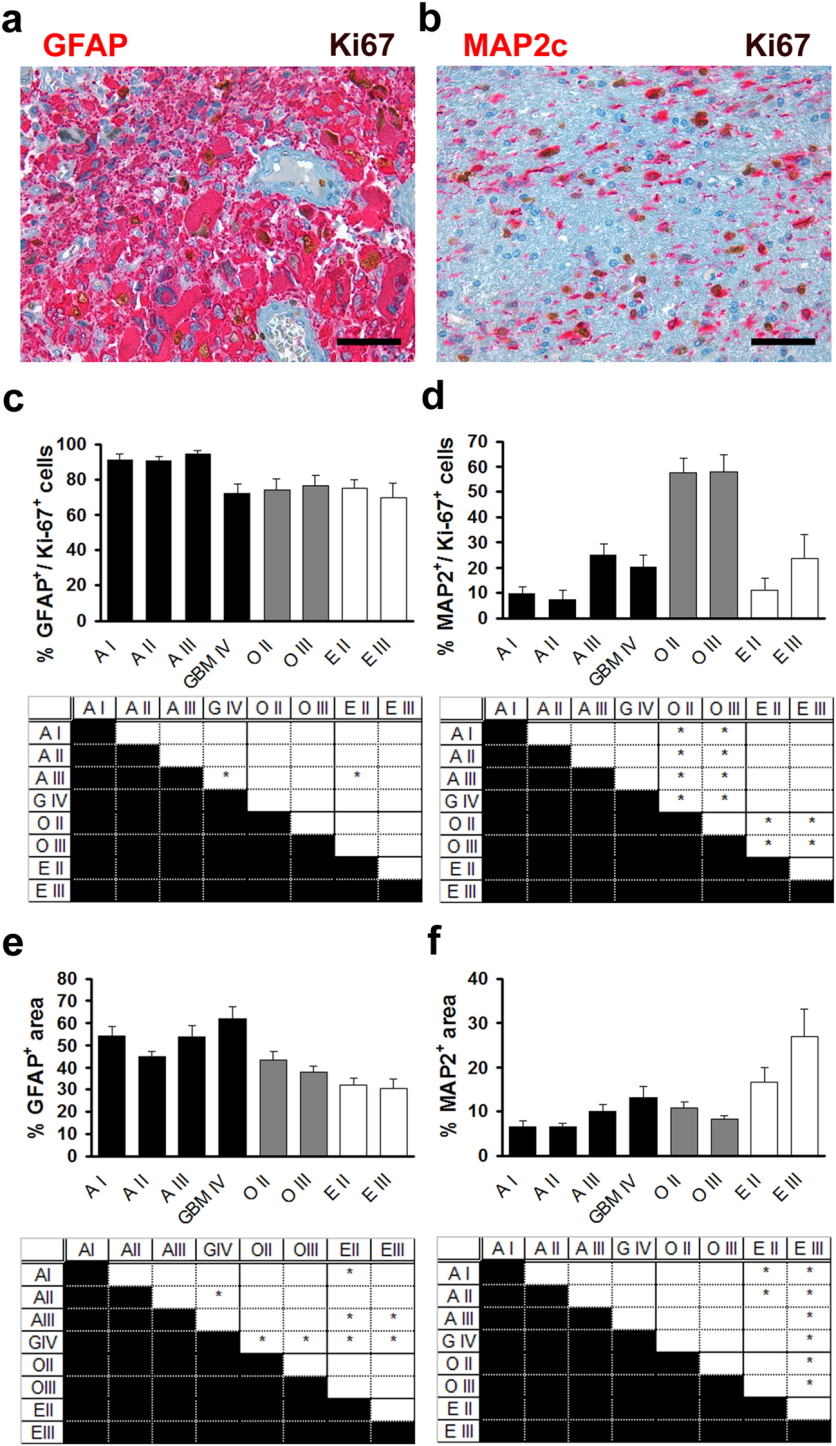
The expression of glial proteins in proliferating tumour cells differs from the expression of glial proteins in non-proliferating tumour cells. Analysis of the expression of GFAP (**a**, **c**, **e**) and MAP2c (**b**, **d**, **f**) in pilocytic astrocytoma WHO grade I (A I), diffuse astrocytoma WHO grade II (A II), anaplastic astrocytoma WHO grade III (A III), glioblastoma WHO grade IV (GBM IV), oligodendroglioma WHO grade II (O II) and WHO grade III (O III), ependymoma WHO grade II (E II), and anaplastic ependymoma WHO grade III (E III). **a** Immunohistochemical double staining with antibodies against Ki-67 (brown) and GFAP (red), scale bar = 100 µm. **b** Immunohistochemical double staining with antibodies against Ki-67 (brown) and MAP2c (red), scale bar = 100 µm. **c** Quantification of the percentage of cells immunopositive for both GFAP and Ki-67 among Ki-67 positive cells in glioma representing the expression of GFAP among proliferating tumour cells. Upper panel shows mean ± s.e.m. Lower panel shows results of one-way analysis of variance (ANOVA) followed by Tukey HSD post hoc test. *Indicates *p* < 0.05. **d** Quantification of the percentage of cells immunopositive for both MAP2c and Ki-67 among Ki-67 positive cells in glioma. Upper panel shows mean ± s.e.m. Lower panel shows results of one-way analysis of variance (ANOVA) followed by Tukey HSD post hoc test. *Indicates *p* < 0.05. **e** Quantification of the percentage of cross-sectional tumour area immunopositive for GFAP representing the expression of GFAP among proliferating and non-proliferating tumour cells. Upper panel shows mean ± s.e.m. Lower panel shows results of one-way analysis of variance (ANOVA) followed by Tukey HSD post hoc test. *Indicates *p* < 0.05. **f** Quantification of the percentage of cross-sectional tumour area immunopositive for MAP2c. Upper panel shows mean ± s.e.m. Lower panel shows results of one-way analysis of variance (ANOVA) followed by Tukey HSD post hoc test. *Indicates *p* < 0.05

We first analysed the expression of GFAP and MAP2, two antigens that are routinely used in the diagnostic procedure for human glioma (Louis et al. 2016). GFAP is an intermediate filament that is expressed by astrocytes in the adult central nervous system (Eng et al. 2000) and glial neoplasms (Ikota et al. 2006). It is expressed by neural stem cells in the mammalian brain during development and in the adult (Doetsch et al. 1999) and by glioma stem cells in cell culture (Gunther, 2008). Immunohistochemical analysis with antibodies against Ki67 and GFAP showed that GFAP was frequently expressed among proliferating cells in all tumour entities with more than 70% of Ki67-positive cells showing GFAP expression in all tumour entities (Fig. 3a, c). There was a tendency towards decreased expression of GFAP in proliferating cells in glioblastoma, oligodendroglioma, and ependymoma versus pilocytic astrocytoma, diffuse astrocytoma, and anaplastic astrocytoma, but the difference amounted to less than 25% and was statistically significant only in anaplastic astrocytoma versus glioblastoma and ependymoma WHO grade II (Fig. 3a, c). In contrast, analysis of total expression of GFAP showed the highest expression in glioblastoma with decreased expression in oligodendroglioma and ependymoma (Fig. 3e) but the increased positivity of GFAP in glioblastoma might be due to higher cell density and not to increased expression of GFAP. Thus, although our results indicate that expression of GFAP may differ between proliferating and non-proliferating cells in glioma, the most remarkable result is that most proliferating cells in all glial neoplasms express GFAP, a marker protein for differentiated astrocytes but also neural stem cells. The immunohistochemical analysis of GFAP reveals a limitation of our evaluation procedure. The measurement of total expression was performed by determining the fraction of the area of immunopositivity of a defined area in each core thus comprising proliferating and non-proliferating cells and might be confounded by residual non-tumoural tissue especially at the periphery of diffusely infiltrating tumours and, therefore, minor differences in marker expression by tumour cells might not reach statistical significance while the analysis of marker-positive proliferating cells was performed by determining the percentage of marker-positive of the Ki67-positive cells. Furthermore, the use of immunohistochemical staining does not allow the analysis of double staining of nuclear antigens like Olig2 together with Ki67. In future studies, immunofluorescence may thus be used which even allows the simultaneous analysis of more than two marker proteins on a single core.

MAP2 is a microtubulus-associated protein that is expressed by neurons in the adult central nervous system (Huber and Matus 1984), glial precursor cells (Blumcke, 2001) in the developing human brain, and by glioma stem cells in cell culture (Gunther et al. 2008). In our study, MAP2 was highly expressed by proliferating cells in oligodendroglial neoplasms with more than 50% of Ki67-positive cells staining positive for MAP2 and significantly lower expression among proliferating cells in astrocytic neoplasms and ependymal neoplasms (Fig. 3b, d). In contrast, the total expression of MAP2 (in proliferating and non-proliferating cells) was not increased in oligodendroglioma. In detail, astroglial and oligodendroglial neoplasms did not significantly differ in MAP2 expression whereas ependymal neoplasms showed a higher expression when compared with both, astrocytic and oligodendroglial neoplasms (Fig. 3f). This is remarkable because it shows that MAP2 is differentially expressed by proliferating and non-proliferating cells in glioma and that oligodendroglioma is characterised by a high percentage of MAP2-positive cells among proliferating cells.

**Fig. 4:**
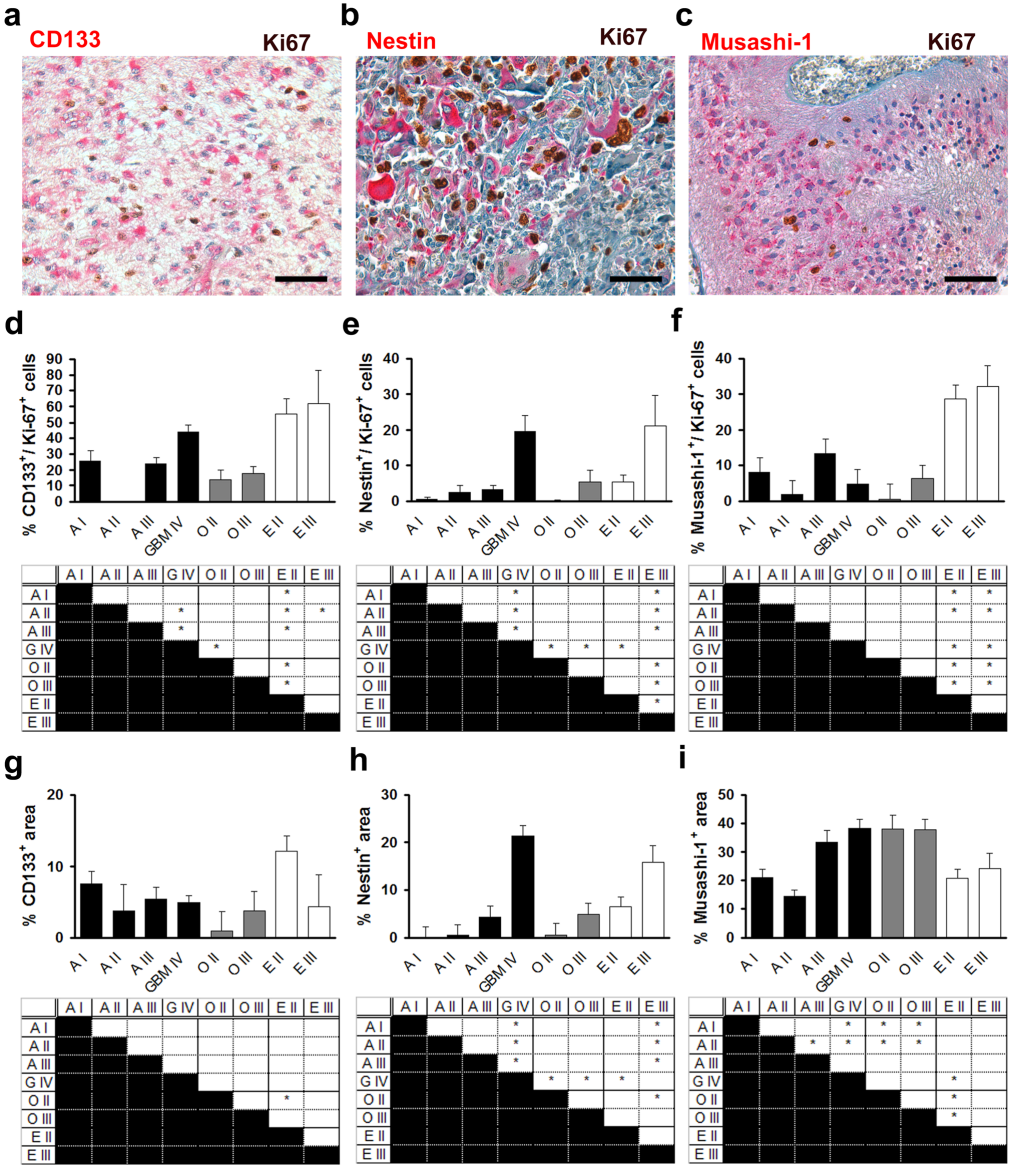
The expression of glioma stem cell markers in proliferating tumour cells differs from their expression in non-proliferating tumour cells. Analysis of the expression of CD133 (**a**, **d**, **g**), Nestin (**b**, **e**, **h**), and Musashi-1 (**c**, **f**, **i**) in pilocytic astrocytoma WHO grade I (A I), diffuse astrocytoma WHO grade II (A II), anaplastic astrocytoma WHO grade III (A III), glioblastoma WHO grade IV (GBM IV), oligodendroglioma WHO grade II (O II) and WHO grade III (O III), ependymoma WHO grade II (E II), and anaplastic ependymoma WHO grade III (E III). **a** Immunohistochemical double staining with antibodies against Ki-67 (brown) and CD133 (red), scale bar = 100 µm. **b** Immunohistochemical double staining with antibodies against Ki-67 (brown) and Nestin (red), scale bar = 100 µm. **c** Immunohistochemical double staining with antibodies against Ki-67 (brown) and Musashi-1 (red), scale bar = 100 µm. **d** Quantification of the percentage of cells immunopositive for both CD133 and Ki-67 among Ki-67 positive cells in glioma representing the expression of CD133 among proliferating tumour cells. Upper panel shows mean ± s.e.m. Lower panel shows results of one-way analysis of variance (ANOVA) followed by Tukey HSD post hoc test. *Indicates *p* < 0.05. **e** Quantification of the percentage of cells immunopositive for both Nestin and Ki-67 among Ki-67 positive cells in glioma. Upper panel shows mean ± s.e.m. Lower panel shows results of one-way analysis of variance (ANOVA) followed by Tukey HSD post hoc test. *Indicates *p* < 0.05. **f** Quantification of the percentage of cells immunopositive for both Musashi-1 and Ki-67 among Ki-67 positive cells in glioma. Upper panel shows mean ± s.e.m. Lower panel shows results of one-way analysis of variance (ANOVA) followed by Tukey HSD post hoc test. *Indicates *p* < 0.05. **g** Quantification of the percentage of cross-sectional tumour area immunopositive for CD133 representing the expression of CD133 among proliferating and non-proliferating tumour cells. Upper panel shows mean ± s.e.m. Lower panel shows results of one-way analysis of variance (ANOVA) followed by Tukey HSD post hoc test. *Indicates *p* < 0.05. **h** Quantification of the percentage of cross-sectional tumour area immunopositive for Nestin. Upper panel shows mean ± s.e.m. Lower panel shows results of one-way analysis of variance (ANOVA) followed by Tukey HSD post hoc test. *Indicates *p* < 0.05. **i** Quantification of the percentage of cross-sectional tumour area immunopositive for Musashi-1. Upper panel shows mean ± s.e.m. Lower panel shows results of one-way analysis of variance (ANOVA) followed by Tukey HSD post hoc test. *Indicates *p* < 0.05

CD133 is a transmembrane glycoprotein that is expressed by embryonic neural stem cells (Pfenninger, 2007) and was the first marker identified for glioma stem cells (Singh et al. 2004). In our study, more than 50% of proliferating cells in ependymal tumours expressed CD133 with significantly lower percentages in tumours of oligodendroglial and astrocytic origin (Fig. 4a, d). In diffuse astrocytic neoplasms (diffuse astrocytoma, anaplastic astrocytoma, and glioblastoma) the percentage of CD133-positive cells among proliferating cells correlated with increasing WHO grade (*r* = 0.406, *p* < 0.01). In contrast, total expression of CD133 (in proliferating and non-proliferating cells) did not differ significantly among astrocytic tumours and between astrocytic, oligodendroglial, and ependymal tumours (Fig. 4 g). Thus, CD133 is differentially expressed by proliferating and non-proliferating cells in glioma. CD133 is debated as a prognostic marker in glioma. Some groups showed that the expression of CD133 and the presence of CD133-positive tumour cells correlated with WHO grade or malignancy in astrocytic tumours (Ma et al. 2008; Thon, 2010) but other groups could not detect significant correlations (Christensen et al. 2008). In our study only CD133 expression by proliferating cells but not total expression of CD133 correlated with WHO grade among astrocytic tumours supporting CD133 as a potential marker for malignancy in astrocytic tumours in combination with other markers. This is supported by a study showing that the presence of tumour cells positive for both, CD133 and Ki67 directly correlated with poor survival in glioblastoma whereas the presence of CD133-positive cells did not (Pallini et al. 2008). On the other hand, our study points towards a context-dependent effect of CD133 on malignancy because the highest expression in proliferating cells was found in ependymal tumours which generally have a more favourable prognosis than diffuse astrocytomas.

Nestin is an intermediate filament that is expressed by neural stem cells in the mammalian central nervous system (Zimmerman, 1994) and has been shown to be expressed by glioma stem cells (Singh et al. 2004). In our study, nestin was expressed in 20% of proliferating cells in glioblastoma WHO grade IV and anaplastic ependymoma WHO grade III with significantly lower expression among proliferating cells in diffuse astrocytoma WHO grade II, anaplastic astrocytoma WHO grade III, oligodendroglioma WHO grade III, and ependymoma WHO grade II and negligible expression in pilocytic astrocytoma WHO grade I and oligodendroglioma WHO grade II (Fig. 4b, e). Thus, the percentage of nestin-expressing cells among proliferating cells increases with increasing WHO grade. Interestingly, the analysis of the total expression of nestin by glioma revealed similar results (Fig. 4h). Thus, no evidence for a differential expression of nestin among proliferating and non-proliferating cells could be found. Our results concerning the total expression of nestin are in line with previous studies showing that nestin expression increased with WHO grade and malignancy (Ma et al. 2008; Wan, 2011). To our knowledge, no previous studies addressed the expression of nestin by proliferating cells in glioma.

Musashi-1 is an RNA-binding protein expressed in neural stem and precursor cells during development and in the adult brain (Kanemura, 2001). In our study, 30% of proliferating cells in ependymal tumours expressed Musashi-1 with significantly lower expression in proliferating cells in astrocytic and oligodendroglial tumours (Fig. 4c, f). In contrast, total expression was highest in oligodendroglial and high grade astrocytic tumours with lower expression in ependymal and low-grade astrocytic tumours (Fig. 4i). Previous studies showed increased numbers of Musashi-1-positive cells in high-grade versus low-grade astrocytic tumours (Ma et al. 2008), higher expression of Musashi-1 in high-grade versus low-grade astrocytic tumours (Thon et al. 2010), and a correlation of the number of Musashi-1-positive cells with grade in astrocytic tumours (Strojnik et al. 2007) confirming our results on total expression of Musashi-1. To our knowledge, no previous studies address the expression of Musashi-1 by proliferating cells in glioma, although one study showed a correlation of Musashi-1 expression and proliferation in glioma (Toda, 2001).

**Fig. 5:**
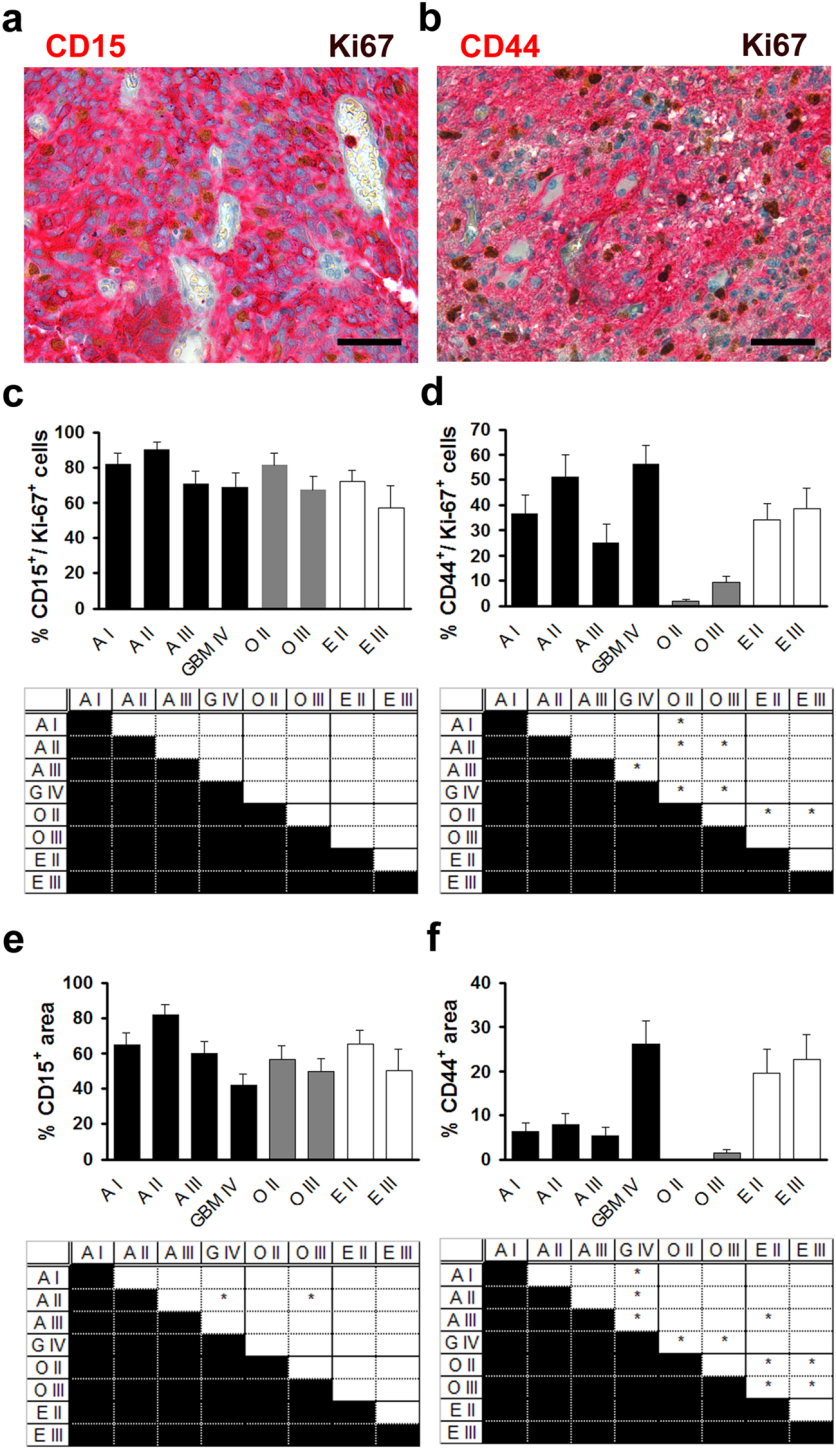
The expression of glioma stem cell markers in proliferating tumour cells differs from their expression in non-proliferating tumour cells (2). Analysis of the expression of CD15 (**a**, **c**, **e**) and CD44 (**b**, **d**, **f**) in pilocytic astrocytoma WHO grade I (A I), diffuse astrocytoma WHO grade II (A II), anaplastic astrocytoma WHO grade III (A III), glioblastoma WHO grade IV (GBM IV), oligodendroglioma WHO grade II (O II) and WHO grade III (O III), ependymoma WHO grade II (E II), and anaplastic ependymoma WHO grade III (E III). **a** Immunohistochemical double staining with antibodies against Ki-67 (brown) and CD15 (red), scale bar = 100 µm. **b** Immunohistochemical double staining with antibodies against Ki-67 (brown) and CD44 (red), scale bar = 100 µm. **c** Quantification of the percentage of cells immunopositive for both CD15 and Ki-67 among Ki-67 positive cells in glioma representing the expression of CD15 among proliferating tumour cells. Upper panel shows mean ± s.e.m. Lower panel shows results of one-way analysis of variance (ANOVA) followed by Tukey HSD post hoc test. *Indicates *p* < 0.05. **d** Quantification of the percentage of cells immunopositive for both CD44 and Ki-67 among Ki-67 positive cells in glioma. Upper panel shows mean ± s.e.m. Lower panel shows results of one-way analysis of variance (ANOVA) followed by Tukey HSD post hoc test. *Indicates *p* < 0.05. **e** Quantification of the percentage of cross-sectional tumour area immunopositive for CD15 representing the expression of CD15 among proliferating and non-proliferating tumour cells. Upper panel shows mean ± s.e.m. Lower panel shows results of one-way analysis of variance (ANOVA) followed by Tukey HSD post hoc test. *Indicates *p* < 0.05. **f** Quantification of the percentage of cross-sectional tumour area immunopositive for CD44. Upper panel shows mean ± s.e.m. Lower panel shows results of one-way analysis of variance (ANOVA) followed by Tukey HSD post hoc test. *Indicates *p* < 0.05

CD15 (Lewis X, stage-specific embryonic antigen 1) is a cell surface protein that is expressed in brain tumours (Reifenberger et al. 1992) and has been suggested to be a cancer stem cell marker (Son et al. 2009). In our study, 60–90% of proliferating tumour cells expressed CD15 (Fig. 5a, c) but no significant differences between tumour entities or correlation with WHO grade were found (Fig. 5c). Similarly, the analysis of total expression of CD15 did not reveal any significant differences between tumour entities or a correlation with WHO grade (Fig. 5e). Thus, no evidence for a differential expression of CD15 among proliferating and non-proliferating cells could be found. These results are in line with a previous study that did not show any correlation of CD15 expression and overall survival in glioblastoma (Kim et al. 2011). To our knowledge, no previous studies address the expression of CD15 by proliferating cells in glioma.

CD44 is a transmembrane glycoprotein that acts as a receptor for hyaluronic acid, is involved in the regulation of proliferation, apoptosis, cell motility, and angiogenesis in inflammation and cancer (Misra et al. 2015). It is a common cancer stem cell marker expressed in various tumours (Brown and Mantamadiotis 2014) and has been suggested as a glioma stem cell marker in Glioblastoma (Fu, 2013; Pietras, 2014). In our study, the expression of CD44 by proliferating cells varied between 20 and 55% in astrocytic tumours including glioblastoma but did not correlate with WHO grade (Fig. 4b, d). In contrast, the total expression of CD44 was significantly higher in glioblastoma versus astrocytoma grade I-III (Fig. 5f) pointing towards a differential expression of CD44 among proliferating and non-proliferating cells in astrocytic tumours. In contrast, no differences in the expression of CD44 were observed between proliferating and non-proliferating cells in oligodendroglioma and ependymoma with high expression in ependymoma grade II and III and negligible expression in oligodendrocytic tumours (Fig. 5f). Our results concerning the total expression of CD44 are in line with a previous study showing a correlation between tumour dignity and CD44 expression in astrocytic tumours including glioblastoma (Wei, 2010). To our knowledge, no previous studies address the expression of CD44 by proliferating cells in glioma.

**Fig. 6:**
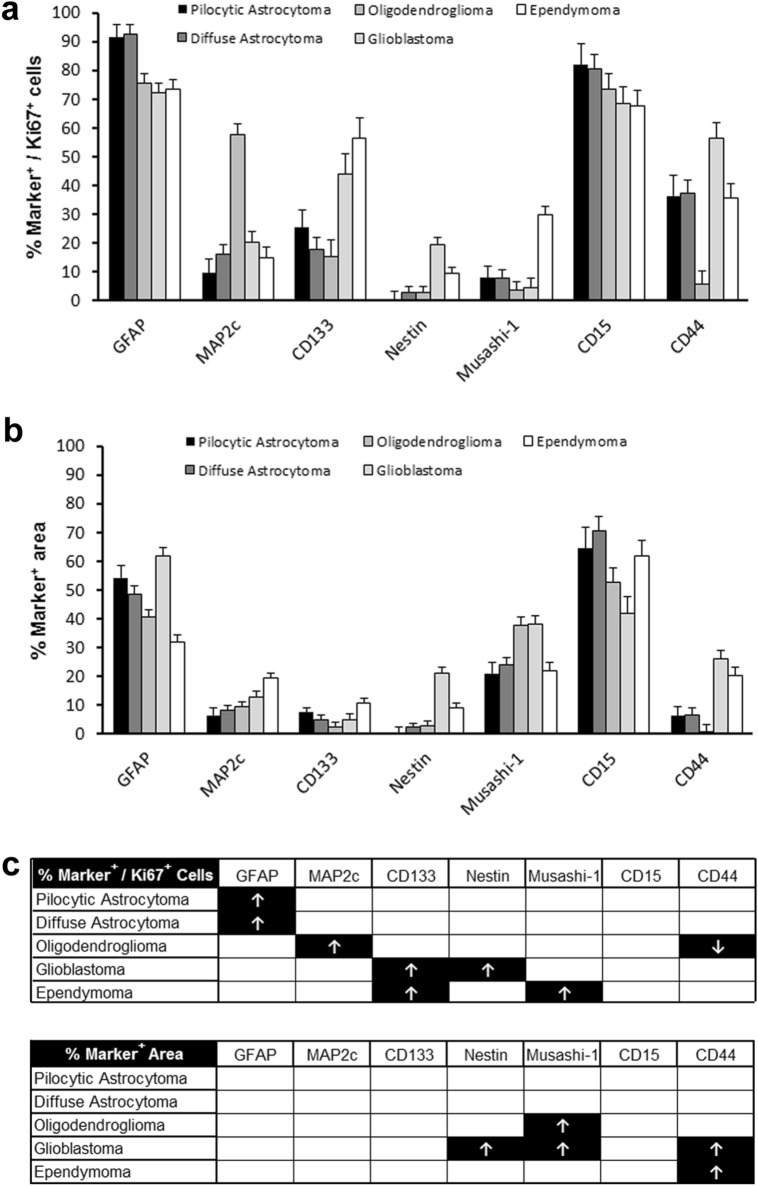
The expression profiles of proliferating and non-proliferating cells differ between astrocytoma, oligodendroglioma and ependymoma. Immunohistochemical analysis of marker expression in proliferating and non-proliferating cells in pilocytic astrocytoma WHO grade I, diffuse astroglial tumours (diffuse astrocytoma WHO grade II and anaplastic astrocytoma WHO grade III), oligodendroglial tumours (oligodendroglioma WHO grade II and anaplastic astrocytoma WHO grade III), glioblastoma WHO grade IV, and ependymal tumours (ependymoma WHO grad II and anaplastic ependymoma WHO grade III). **a** Quantification of the percentage of cells immunopositive for both Ki-67 and different marker proteins among Ki-67 positive cells in different glial tumour entities representing marker expression among proliferating tumour cells (mean ± s.e.m.). **b** Quantification of the percentage of cross-sectional marker-positive tumour area representing marker expression among proliferating and non-proliferating tumour cells (mean ± s.e.m.). **c** Results of one-way analysis of variance (ANOVA) followed by Tukey HSD post hoc test. Arrows indicate significantly (*p* < 0.05) increased (↑) or decreased (↓) values in comparison with tumour entities not marked with arrows

Thus, the expression of both glial and glioma stem cell markers differs between proliferating and non-proliferating cells in glioma. Furthermore, marker expression of proliferating cells differs between different brain tumour entities. Figure 6 summarises these differences between the tumour entities (Fig. 6a, b, c). In these analyses, astrocytomas grade II and III were grouped together as diffuse astrocytoma and oligodendrogliomas grade II and III were grouped together as oligodendroglioma. Whereas the expression of GFAP was higher in proliferating cells in pilocytic and diffuse astrocytoma when compared with other brain tumour entities, proliferating cells in oligodendroglioma were characterised by a higher expression of MAP2 and a lower expression of CD44. In contrast, proliferating cells in both, glioblastoma and ependymoma showed a higher expression of CD133, but proliferating cells in glioblastoma furthermore showed a higher expression of Nestin whereas proliferating cells in ependymoma showed a higher expression of Musashi-1. Thus, proliferating cells in different tumour entities showed different expression profiles. Interestingly, the expression profiles of proliferating cells in pilocytic astrocytoma and diffuse astrocytoma did not significantly differ. This is puzzling because pilocytic astrocytoma and diffuse astrocytoma differ significantly in their prognosis (Louis et al. 2016). A more extended analysis including more marker proteins might reveal differences between pilocytic and diffuse astrocytoma in future studies.

In summary, our study shows, that the expression of glial and glioma stem cell markers differs between proliferating and non-proliferating cells in glioma and that the proliferating cells in different glial tumour entities show distinct expression profiles. The authors are aware of the fact that this is an initial study only. Nevertheless, our study emphasises the importance of thoroughly characterising proliferating cells in glioma and other tumours. Future studies, especially involving immunofluorescence will allow the extension of the analyses to nuclear antigens and the analysis of the expression of multiple antigens in single proliferating cells with the potential to define subgroups of proliferating cells. Furthermore, molecular pathways involved in proliferation, cell survival and apoptosis that can potentially be targeted could be analysed.

Thus, further analysis of the expression profile of proliferating cells in glioma and correlation with clinical parameters like outcome and susceptibility to therapy might help establish new predictive and prognostic biomarkers and open up new therapeutic options for glial tumours.

## Data Availability

Not applicable.
